# Tribological Properties of Water-lubricated Rubber Materials after Modification by MoS_2_ Nanoparticles

**DOI:** 10.1038/srep35023

**Published:** 2016-10-07

**Authors:** Conglin Dong, Chengqing Yuan, Lei Wang, Wei Liu, Xiuqin Bai, Xinping Yan

**Affiliations:** 1 Key Laboratory of Marine Power Engineering & Technology (Ministry of Transport), Wuhan University of Technology, Wuhan 430063, P. R. China; 2 State Key Laboratory of Tribology, Tsinghua University, Beijing 100084, China; 3 China Ship Development and Design Center, Wuhan 430064, P. R. China

## Abstract

Frictional vibration and noise caused by water-lubricated rubber stern tube bearings, which are generated under extreme conditions, severely threaten underwater vehicles’ survivability and concealment performance. This study investigates the effect of flaky and spherical MoS_2_ nanoparticles on tribological properties and damping capacity of water-lubricated rubber materials, with the aim of decreasing frictional noise. A CBZ-1 tribo-tester was used to conduct the sliding tests between rubber ring-discs and ZCuSn_10_Zn_2_ ring-discs with water lubrication. These materials’ typical mechanical properties were analysed and compared. Coefficients of friction (COFs), wear rates, and surface morphologies were evaluated. Frictional noise and critical velocities of generating friction vibration were examined to corroborate above analysis. Results showed that spherical MoS_2_ nanoparticles enhanced rubber material’s mechanical and tribological properties and, in turn, reduced the friction noise and critical velocity. Flaky MoS_2_ nanoparticles reduced COF but did not enhance their mechanical properties, i.e., the damping capacity, wear resistance property; thus, these nanoparticles did not reduce the critical velocity obviously, even though increased the frictional noise at high load. The knowledge gained in the present work will be useful for optimizing friction pairs under extreme conditions to decrease frictional noise of water-lubricated rubber stern tube bearings.

Water-lubricated rubber stern tube bearings are important supporting parts of the propulsion systems of underwater vehicles (e.g., submarines). When submarines are in states of low speed, heavy load, starting or stopping, it is very difficult to form an effective water-lubricated film layer between the rubber stern tube bearings and stern shafts. Hence, mixed lubrication, boundary lubrication, or dry friction conditions occur^1^,^2^,^3^. In these cases, the temperature in local areas of the wear surface of the rubber stern tube bearings increases sharply because of its poor heat conductivity and heat generated during the wear process^4^,^5^. As a result, the friction and wear increase in severity. The stick-slip phenomenon can be easily generated and eventually results in frictional vibration and radiating noise, severely undermining the submarine’s survivability and concealment performance. When the linear velocity of the stern shaft is less than 0.6 m/s, the frictional vibration is generated on the surface where the rubber stern tube bearing contacts the stern shaft, thus radiating frictional noise^6^. Reports have revealed that this reduced the radiatednoise of underwater vehicles by 6 decibels and the working distance of sonar by 50%^7^. Therefore, solving this problem is very important to increase underwater vehicles’ survivability and reduce environmental noise pollution.

Reducing the frictional vibration and increasing the damping capacity of rubber materials are very effective methods for decreasing and controlling frictional noise. Hence, researchers have attempted to modify rubber materials to obtain a good self-lubrication property to reduce the frictional vibration^8^. Molybdenum disulphide (MoS_2_) is a good solid lubrication additive and has been known as the “the king of lubrication” for a long time because of its excellent self-lubrication property^9^. Therefore, MoS_2_ is frequently used to improve the self-lubrication properties of rubber. To date, various preparation methods, which includ thermal reduction, high-temperature sulfurization, hydrothermal methods, chemical vapour deposition (CVD), and even laser ablation, have been developed to produce different types of MoS_2_ nanoparticles, for example, different specific surface areas and geometric constructions^10^,^11^,^12^,^13^,^14^,^15^. However, the identification of MoS_2_ nanoparticles suitable for use as lubricant additives for rubber materials to improve both the materials’ self-lubrication properties and damping capacities remains an open research topic. Nitrile butadiene rubber (NBR), a polymer, is a well-known organic macromolecular compound that is elastic and highly resistant to fatigue and wear. Moreover, it absorbs vibrations and has excellent chemical stability^16^,^17^. Therefore, NBR-type materials have been widely used to fabricate water-lubricated rubber stern tube bearings for marine applications. This paper choose the flaky and spherical MoS_2_ nanoparticles (Fig. 1a,b) as solid lubricant additives for the NBR material, and study the effects of different types of MoS_2_ nanoparticles on water-lubricated rubber materials’ tribological properties and damping capacities, with the goal of decreasing and controlling frictional noise. Different types of rubber specimens were tested against ZCuSn_10_Zn_2_ discs with a disc tribo-tester. For convenience, the rubber materials with no MoS_2_ nanoparticles, with flaky MoS_2_ nanoparticles, and with spherical MoS_2_ nanoparticles were named NBR, NBR-FMS, and NBR-SMS, respectively. Figure 1c shows the x-ray diffraction patterns of the three materials. NBR-FMS and NBR-SMS showed the obvious peaks of 2θ = 14.1° which were the characteristic diffraction peaks of MoS_2_, and revealed that the MoS_2_ nanoparticles kept the original layer structure in the rubber materials^18^,^19^,^20^.

## Results

### Analysis of mechanical properties

Shore hardness, tensile strength, tear strength and compressive property are not only important parameters for the characterization of rubber materials’ mechanical properties but also can affect its tribological properties significantly^21^,^22^. In general, the service temperatures of ship water-lubricated stern tube rubber bearing are usually from 0 °C to 80 °C. Therefore, the shore hardness, tensile strength, and tear strength as a function of temperature from 0 °C to 80 °C were examined as viewed in Fig. 2a–c. In general, these properties decreased as the measurement temperature increased, and the decreasing trends became steeper as the temperature increased from 40 °C to 80 °C. As shown in Fig. 2a, at the same temperature, the shore hardness exhibited the following order: NBR-FMS > NBR-SMS > NBR. The elastic modulus of MoS_2_ was much larger than that of the rubber, and thus, the addition of the MoS_2_ nanoparticles imbued the rubber materials with a large shore hardness. The tensile strength of NBR-SMS was much larger than those of the other two, whereas the tensile strengths of NBR and NBR-FMS were similar, as shown in Fig. 2b. Figure 2c indicates that the tear strength of NBR-SMS exceeded that of NBR, whereas the tear strength of NBR-FMS was smaller than that of NBR. Figure 2d shows the dynamic compressive properties of the three rubber materials at the compressive strain rate of 2640 s^−1^ and room temperature. Generally, the compressive stresses increased almost linearly when the compressive strain was lower than 30%, but their increasing trends became steep obviously as the compressive strain increased further. As the details, at the same compressive strain, the compressive stresses exhibited the following order: NBR-FMS > NBR-SMS > NBR. The test data obviously revealed that the rough, spherical MoS_2_ nanoparticles enhanced the rubber material’s tensile strength and tear strength, whereas the smooth, flaky MoS_2_ nanoparticles did not, and sometimes even reduced them. However, flaky MoS_2_ nanoparticles enhanced the shore hardness and compressive property more obviously compared with spherical MoS_2_ nanoparticles because of their geometric structure, and these phenomena were consistent with the Eshelby’s inclusion theory^23^,^24^.

Storage modulus, loss modulus, and loss factor characterize the viscoelasticity of a rubber and reflect its damping properties effectively. Figure 3 shows the effects of MoS_2_ nanoparticles on the dynamic mechanical analysis (DMA) patterns of the three rubber materials at service temperatures from 0 °C to 80 °C. The storage modulus decreased sharply as the temperature increased, especially in the range from 0 °C to 30 °C as shown in Fig. 3a. However, the storage moduli of NBR-FMS and NBR-SMS were larger than that of NBR at the same temperature. Figure 3b shows that the loss modulus of NBR-SMS was significantly larger than those of NBR and NBR-FMS in the range from 0 °C to 60 °C. Thus, NBR-SMS absorbed more mechanical energy under dynamic deformation. The loss factor is the ratio of the loss modulus and the storage modulus, i.e., Tan *δ* = *E”*/*E’*. The peak of the loss factor was the highest for NBR-SMS (0.71), as shown in Fig. 3c, and shifted to a markedly higher temperature. Moreover, it remained at a good value throughout the wide temperature range from 0 °C to 67 °C. The peak of the loss factor of NBR-FMS was the smallest (0.64) and was shifted to a slightly higher temperature compared with that of NBR. The test data suggested that the damping capacity of NBR-SMS was the best and that the spherical MoS_2_ nanoparticles more reinforced the loss factor compared with the flaky MoS_2_ nanoparticles.

Payne effect refers to the effect of strain-dependence on the dynamic viscoelastic properties of filled amorphous polymers, and indirectly evaluates the effects of the fillers on rubber material’s microstructure^25^,^26^. The three rubber materials’ Payne effects were examined, as shown in Fig. 4. Figure 4a indicates that the storage modulus (*E’*) of NBR-SMS changed slightly when the strain amplitudes were less than 15% but decreased sharply (by 84 MPa) as the strain increased (from 15% to 120%). The storage modulus of NBR-FMS was less strain-dependent in the low-strain region (<5%), but an obvious strain-dependent behaviour was observed at higher strains, i.e., a decrease of 117 MPa (from 174 MPa to 57 MPa) occurred. NBR exhibited less strain-dependence than NBR-FMS and NBR-SMS, and its storage modulus decreased by 43 MPa. The loss moduli of NBR and NBR-SMS showed pronounced peaks, as shown in Fig. 4b, and the peak of NBR-SMS shifted to a higher strain compared with NBR-FMS. However, the variation of the loss modulus influenced the loss factor only slightly because of the substantial decrease in the storage modulus, which increased the value of tan *δ* accordingly, as shown in Fig. 4c. These observations suggested that the spherical MoS_2_ nanoparticles reinforced the rubber’s microstructure at high deformation amplitudes compared with flaky MoS_2_ nanoparticles.

### Analysis of COF

The average COFs between the rubber ring-discs and ZCuSn_10_Zn_2_ ring-discs are shown in Fig. 5. The average COFs were negatively correlated with the sliding velocities, decreasing rapidly when the velocities ranged from 0.055 to 0.44 m/s, slowly in the range of 0.44–1.1 m/s and remaining steady when the sliding velocities exceeded 1.1 m/s. In all cases, the trends of the COF relative to the sliding velocities conformed to the Stribeck curve. Thus, the rubber ring-disc and ZCuSn_10_Zn_2_ disc pairs experienced boundary lubrication, mixed lubrication, and hydrodynamic lubrication. Many researchers have obtained similar results^27^,^28^. Under the boundary and mixed lubricated conditions, the COFs of NBR-FMS and NBR-SMS were much lower than that of NBR, and the COF of NBR-SMS was slightly higher than that of NBR-FMS. Therefore, the MoS_2_ nanoparticles significantly improved the self-lubrication properties of the rubber material, and the smooth flaky MoS_2_ nanoparticles better improved the lubrication conditions compared with the rough spherical MoS_2_ nanoparticles. At higher sliding velocities (exceeding 1.1 m/s), the COFs of the three rubber materials were similar.

### Analysis of distance wear volume

Figure 6 shows the behaviours of the distance wear volumes under the sliding distance of 1 km for the three rubber materials as a function of the sliding velocity. Generally, the average distance wear volumes decreased as the velocity increased, sharply in the range of 0.055~0.44 m/s and slowly in the range of 0.44~1.1 m/s. The distance wear volumes were almost unchanged when the velocity exceeded 1.1 m/s. Notably, the distance wear volumes of NBR-FMS were larger than those of NBR-SMS, and even were larger than those of NBR at 0.055 and 0.11 m/s as shown in the inset, whereas the distance wear volumes of NBR-SMS were smaller than that of NBR. Obviously, the flaky MoS_2_ nanoparticles went against for reinforcing the wear-resisting property compared with the spherical MoS_2_ nanoparticles at low velocities. When the velocity was 0.22~1.1 m/s, the distance wear volumes exhibited the following trend: NBR > NBR-FMS > NBR-SMS.

### SEM analysis of the worn surface topographies

To highlight the typical wear characteristics, which were subjected to boundary lubrication, mixed lubrication, and hydrodynamic lubrication conditions, this section presents the SEM images of their wear surfaces at three sliding velocities, i.e., 0.055, 0.55 and 3.3 m/s.

As shown in Fig. 7, many highly deformed NBR asperities, which resulted from the ielded plastic deformation, were visible on the worn surface at 0.055 m/s; moreover, some small tensile phenomena also occurred. As the velocity increased, the deformed NBR asperities became smaller (Fig. 7b). Although wear tracks could be seen on the wear surface at 3.3 m/s, no deformed NBR asperities could be identified (Fig. 7c).

Many highly deformed NBR-FMS asperities were evident on the wear surface in Fig. 8a. Additionally, a hole appeared at the bottom of the deformed NBR-FMS asperities. Furthermore, a flaky particle was extruded from the hole during the wear process. This particle was investigated by Raman spectroscopy, which revealed characteristic peaks at 379 cm^−1^ and 405.6 cm^−1^ (see Fig. 8a). These peaks were very close to the standard characteristic peaks of MoS_2_ at 380 cm^−1^ and 404 cm^−1^. Thus, the flaky particle was a flaky MoS_2_ particle. The wear surface had yielded plastic deformation and accumulation (see Fig. 8b). A hole was clearly seen, and a particle, which was proven to be a MoS_2_ particle, was also present. The stretching and tearing phenomena of the rubber material were apparent above the MoS_2_ particle. Figure 8c shows that no special wear characteristics were present on the wear surface.

The SEM images of the tested NBR-SMS ring-discs at velocities of 0.055, 0.55, and 3.3 m/s are presented in Fig. 9. The deformation phenomenon was not highly apparent for NBR-SMS at 0.055 and 0.55 m/s, although wear tracks were clearly observed. There were no obvious characteristics on the wear surfaces at 3.3 m/s.

Generally, because of the frictional forces and tensile stresses, the three rubber materials experienced deformation, tensile, and tearing during the wear process, and were similar to the typical wear characteristics of a rubber material^29^,^30^. The flaky and spherical MoS_2_ nanoparticles strongly influenced the rubber material’s tribological properties, especially under low-velocity conditions.

### Analysis of frictional noise

The frictional noise and critical velocities (at which the rubber ring-disc specimens began to generate frictional vibrations) were examined under different applied loads at room temperature to verify the above analysis and determine whether NBR-FMS and NBR-SMS could effectively reduce the frictional noise.

Frictional vibration characteristics were used to determine the critical velocities of beginning to generate the frictional vibrations of the three rubber materials as a function of load as shown in Fig. 10a. The applied load strongly affected the critical velocities, which increased the critical velocity as the load increased. The critical velocities of NBR-FMS and NBR-SMS were smaller than that of NBR under the same load, and NBR-SMS’s critical velocity was the lowest. Under heavy loads (0.7 MPa and 0.9 MPa), the critical velocities of NBR-FMS were slightly smaller than those of NBR, whereas NBR-SMS’s critical velocities were much smaller than those of NBR and NBR-FMS. Under the normal load (0.3 MPa) and 0.22 m/s, the frictional noises exhibited the following order: NBR > NBR-FMS > NBR-SMS as viewed in Fig. 10b, whereas exhibited the following trend: NBR-FMS > NBR > NBR-SMS under the high load (0.9 MPa). Figure 10c,d show the frequency-domain spectrum characteristics of the frictional vibrations when the three rubber materials generated the frictional noise as shown in Fig. 10b. Obviously, the relative vibrational acceleration at 1.24 KHz of NBR was the biggest at 0.3 MPa, and NBR-SMS’s relative vibrational acceleration was the smallest. Under 0.9 MPa condition, the relative vibrational acceleration at 1.32 KHz of NBR-FMS was higher than those of NBR and NBR-SMS, and moreover, the vibrations at the frequencies of 0.14, 0.3 and 5.23 KHz occurred. These phenomena indirectly reflected that the friction and wear on NBR-FMS’s wear surface increased in severity. Whereas, NBR-SMS still kept the vibration with the smallest relative vibrational acceleration at 1.39 KHz. The frictional vibration characteristics were consistent with the frictional noise results. These data indicated that NBR-SMS had a strong ability to reduce the frictional noise and critical velocity as compared with NBR-FMS and NBR, particularly under high load conditions. NBR-FMS could reduce the critical velocity slightly, but increased the frictional noise under the high load.

## Discussion

### Influence of flaky and spherical MoS_2_ nanoparticles on mechanical properties

MoS_2_ is chemically inert, i.e., not reactive with rubber molecules. The MoS_2_ nanoparticles mainly adhere to the rubber molecules through van der Waals forces^31^,^32^,^33^. The test results, which were shown in Figs 7, 8, 9, revealed that the tension process might play an important in the wear process. Thus, the tension model of the MoS_2_ nanoparticles and rubber material is shown in Fig. 11. The MoS_2_ nanoparticles exhibit almost no deformation compared with the rubber material under the same load because of the nanoparticles’ large elastic modulus. Inserts I and II in Fig. 11a show that the rubber in contact with the surfaces of flaky MoS_2_ nanoparticles exhibited clear deformation along the stress direction when NBR-FMS underwent stretching or tearing deformation, which resulted in relative displacement between the contact interfaces of the rubber and MoS_2_ nanoparticles. Interfacial debonding between the rubber and flaky MoS_2_ nanoparticles could reasonably occur because of the low binding force and relative displacement, leading to the generation of defects in the rubber material, particularly in the high-strain region. Consequently, the flaky MoS_2_ nanoparticles exhibited a clear strain-dependence and exerted limited effects on improving the tensile strength and tear strength of the rubber material, even reducing these properties in some cases as shown in Figs 2b,c and 4. Additionally, the smooth flaky MoS_2_ nanoparticles, which were good solid lubricant, contributed to improving the interface lubrication. As a result, the internal friction of NBR-FMS decreased under the same dynamic load and exhibited a reduced loss modulus and loss factor (Fig. 3b,c).

The surface with a sheet-like structure of spherical MoS_2_ nanoparticles was very rough. When the spherical MoS_2_ nanoparticles embedded in the rubber material, the rubber material also embedded into the valley floor of the rough surface and made strong contacts with the nanoparticles, thus increasing the adhesion force. Additionally, the rough surface prevented the embedded rubber material from stretching or tearing effectively, locked the materials in the valley floor, as shown in Fig. 11b. Hence, the large adhesion force and rough surface reduced the relative displacement and interfacial debonding phenomena, which resulted in a decreased number of defects. It is reasonable to suggest that the spherical MoS_2_ nanoparticles enhanced the tensile strength and tear strength and reduced the Payne effect of NBR-SMS^34^,^35^. Additionally, the rough spherical MoS_2_ nanoparticle surface increased the internal friction, eventually reinforced the loss modulus and loss factor. Thus, the rubber material with added spherical MoS_2_ nanoparticles had a good damping capacity compared with NBR.

### Influence of flaky and spherical MoS_2_ nanoparticles on lubrication properties

Undoubtedly, the flaky and spherical MoS_2_ nanoparticle additives had played a very important role in reducing the COF of the rubber materials under boundary and mixed lubrication conditions. The layered and low shear strength properties of MoS_2_ lead to excellent self-lubrication property^36^. Thus, part of MoS_2_ nanolayers must be easily sheared and transferred to the counterpart’s wear surface. To prove this phenomena and explain the lubrication mechanisms of the flaky and spherical MoS_2_ nanoparticles in the rubber material, the compositions of ZCuSn_10_Zn_2_ ring-discs’ wear surfaces, which were tested against the three rubber materials at 0.055 m/s and 0.3 MPa, were examined using Raman spectroscopy and energy dispersive spectrometer (EDS), and the results were shown in Fig. 12a,b. Raman analysis revealed that the wear surfaces of ZCuSn_10_Zn_2_ ring-discs, which were tested against NBR-FMS and NBR-SMS, presented the obvious characteristic peaks (379.5 cm^−1^ and 404.5 cm^−1^) around 380 cm^−1^ and 404 cm^−1^ belonged to MoS_2_. As a reference, there was no characteristic peak for the ring-disc’s wear surfaces tested against NBR. The test data fully proved that part of MoS_2_ nanolayers had been transferred to the ring-disc’s wear surfaces. EDS analysis, as viewed in Fig. 12b, showed the element contents of S and Mo to quantitatively analyze the contents of MoS_2_ on the ring-discs’ wear surfaces to further verified the transferring phenomena of MoS_2_. The element contents of S and Mo on the ring-disc’s wear surface tested against NBR-FMS were 4.12% and 5.74% respectively. They were significantly higher than the element contents of S(3.06%) and Mo(4.17%) on the ring-disc’s wear surface tested against NBR-SMS, and were about 34.6% and 37.6% higher respectively. EDS analysis directly proved that it is easier to be sheared and transferred for flaky MoS_2_ nanoparticles compared with spherical MoS_2_ nanoparticles. These differences were the key factors to reasonability reflect the different lubrication properties for NBR-FMS and NBR-SMS as shown in Fig. 5.

Figure 12c presents the lubrication mechanism model of the flaky MoS_2_ nanoparticles in the NBR-FMS during the wear process. When the NBR-FMS contacted with the ZCuSn_10_Zn_2_ ring-disc, the flaky MoS_2_ nanoparticles were bulged on the wear surface and easily formed a surface contact with the ring-disc. In that case, the layered and low shear strength properties of MoS_2_ were helpful to reduce the COF. Moreover, the MoS_2_ nanolayers, which were sheared during the wear process, were transferred to the ZCuSn_10_Zn_2_ ring-disc’s wear surface, and formed a layer of MoS_2_ film and consequently, reduced the COF further. This lubrication mechanism made the flaky MoS_2_ nanoparticles had the best self-lubrication property for the rubber materials under low velocities as shown in Fig. 5. However, it is difficult to form the surface contact between the spherical MoS_2_ nanoparticles and ring-disc because of its geometrical shape as shown in Fig. 12d. As the wear process increased, the MoS_2_ nanosheets, which embedded in the surface of the spherical MoS_2_ nanoparticles, were peeled off and transferred to ring-disc’s wear surface and eventually, formed a layer of MoS_2_ film composed of nanosheets, and improved the lubrication condition significantly. As a result, the COF was reduced effectively. Obviously, it is not easy that the nanosheets on the spherical MoS_2_ nanoparticles’ surfaces were transferred to the ring-disc’s wear surface compared with flaky MoS_2_ nanoparticles, which could be proved by the differences of the element contents of S and Mo in Fig. 12b, and made lower decrease of COF compared with flaky MoS_2_ nanoparticles under the low velocities as viewed in Fig. 5.

At higher sliding velocities (exceeding 1.1 m/s), more water was brought to the media interface, and the lubrication conditions were greatly improved because of elasto-hydrodynamic lubrication, thus facilitated the formation of a thick water-lubricated film that prevented contact between the rubbing pairs and reduced the COF sharply. In this case, the good self-lubrication properties of the rubber materials, which were enhanced by the MoS_2_ nanoparticles, did not substantially contribute to decreasing the COF and consequently, the COFs of the three rubber materials were similar as shown in Fig. 5.

### Influence of flaky and spherical MoS_2_ nanoparticles on wear-resisting properties

The wear process experienced boundary lubrication at 0.055 and 0.11 m/s. The friction forces between the two media were high and the wear process worsened, which resulted in relatively appearance of the yielded plastic deformations as shown in Fig. 8a. The flaky MoS_2_ nanoparticles on the wear surface easily fell off because of the poor binding force and deformation during the wear process. This phenomenon caused defects and resulted in increasing the distance wear volumes (Fig. 6), although the self-lubrication properties of NBR-FMS were better than those of NBR and NBR-SMS. Overall, the flaky MoS_2_ nanoparticles went against reducing the wear resistance under high loading; similar results have been reported^37^. However, the rough surface of the spherical MoS_2_ nanoparticles improved the self-lubrication properties and effectively enhanced the tensile strength and tear strength of NBR-SMS (Fig. 2b–d), thereby reducing the distance wear volume and deformation phenomena, as shown in Figs 6 and 9a. These effects may be the reason why the distance wear volume of NBR-SMS was the smallest. As the velocity increased, the lubrication clearly improved, thus reducing the friction force (see the COF in Fig. 5), and consequently, the nanoparticles did not fall off from the wear surface easily and were able to still contribute to improve the self-lubrication properties of NBR-SMS and NBR-FMS. As a result, the distance wear volumes of NBR-SMS and NBR-FMS were smaller than that of NBR, as shown in Fig. 6. When the sliding velocity further increased, because the rubbing pairs were rarely in contact with each other, the distance wear volumes of the three rubber materials were low, and no obvious characteristics were evident on their wear surfaces.

### Influence of flaky and spherical MoS_2_ nanoparticles on frictional noise

As well known, the frictional vibration is one of the main reasons to generate frictional noise, and frictional force is the exciting force of the frictional vibration. The damping capacity characterizes the capacity of absorbing the mechanical energy under dynamic deformation^38^,^39^. Reducing the frictional force and increasing the damping capacity of rubber materials are very effective methods for decreasing and controlling the frictional noise.

To further explain the effects of the MoS_2_ nanoparticles on the frictional noise for rubber materials, the friction forces and distance wear volumes of the three rubber materials under the low and high loads (0.3 and 0.9 MPa) at the velocity of 0.22 m/s had been examined, and the results were presented in Fig. 13. Under the low load, the NBR-FMS and NBR-SMS reduced the friction forces obviously as shown in Fig. 13a, and eventually resulted in smaller the frictional noises and relative vibrational accelerations compared with NBR as presented in Fig. 10b,c. Gained the biggest average loss factor as viewed in Fig. 13c, NBR-SMS had the largest decrease of frictional noise. The wear resistance property of NBR-FMS was challenged under high load (0.9 MPa), made the NBR-FMS’s distance wear volume was much higher than those of NBR and NBR-SMS (Fig. 13b), which was consistent with the distance wear volume results (Fig. 6) and relative discussions. This phenomenon reflected the friction and wear on the NBR-FMS’s wear surface increased severely, and destroyed the wear and lubrication states, which resulted in increasing the frictional force compared with NBR-SMS as viewed in Fig. 13a, even though NBR-FMS’s self-lubrication property was better than that of NBR-SMS (Fig. 5). Moreover, NBR-FMS’s average loss factor was much lower than that of NBR-SMS and NBR. Hence, it had the worst damping capacity of reducing the frictional vibration, and consequently, both of NBR-FMS’s frictional noise and relative vibrational acceleration were the greatest among them. With good self-lubrication property and big average loss factor, NBR-SMS could reduce the frictional force and frictional vibration, and still decreased the relative vibrational acceleration and frictional noise obviously. In all, the flaky and spherical MoS_2_ nanoparticles had great influences on the self-lubrication property, wear resistance property and damping capacity of the rubber material, regulated the rubber materials’ loss factor and friction force during the wear process, and eventually resulted in differences of the critical velocities and frictional noise. The flaky MoS_2_ nanoparticles reduced the COF but did not effectively enhance the material’s damping capacity and wear resistance property. Thus, these nanoparticles did not reduce the critical velocity of frictional vibration observably at the same load (Fig. 10a), even though increased the frictional noise at high load. In contrast, the spherical MoS_2_ nanoparticles enhanced the mechanical and tribological properties of the rubber material and eventually reduced its critical velocity and frictional noise.

The critical velocity of frictional vibration is a very important parameter to examine the noise reduction capability of rubber materials. Therefore, for future studies, the critical velocity of frictional vibration and stick-slip phenomenon of rubber materials will be investigated to meticulously and deeply. Outcomes will be reported in due course.

### Conclusions

This study investigated the tribological properties of rubber materials and the effects of MoS_2_ nanoparticles on these properties. The typical mechanical and tribological properties of the rubber materials were investigated through comparative analyses of the shore hardness, tensile strength, tear strength, compressive property, DMA, COF, distance wear volume, worn surface topographies, frictional noises and critical velocities under different test conditions. The tribological properties of the rubber material differed significantly depending whether they contained flaky or spherical MoS_2_ nanoparticles. The results showed that the spherical MoS_2_ nanoparticles enhanced the rubber material’s mechanical and tribological properties, eventually reducing its frictional noise. The flaky MoS_2_ nanoparticles reduced the COF but could not enhance the rubber material’s mechanical properties, including the damping capacity, tensile strength, and tear strength; thus, these nanoparticles did not reduce the critical velocity of frictional vibration observably, even though increased the frictional noise at high load. The knowledge gained in this study will likely contribute to optimizing the friction pairs to decrease and control the frictional noise of water-lubricated rubber stern tube bearings.

## Methods and Experiments

### Experimental materials

**Figure 1:**
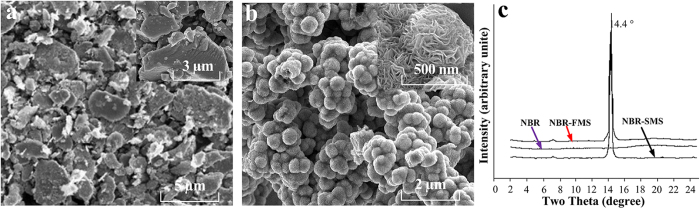
SEM images of (**a**) flaky MoS_2_ nanoparticles and (**b**) spherical MoS_2_ nanoparticles, (**c**) x-ray diffraction patterns of the three materials.

**Figure 2:**
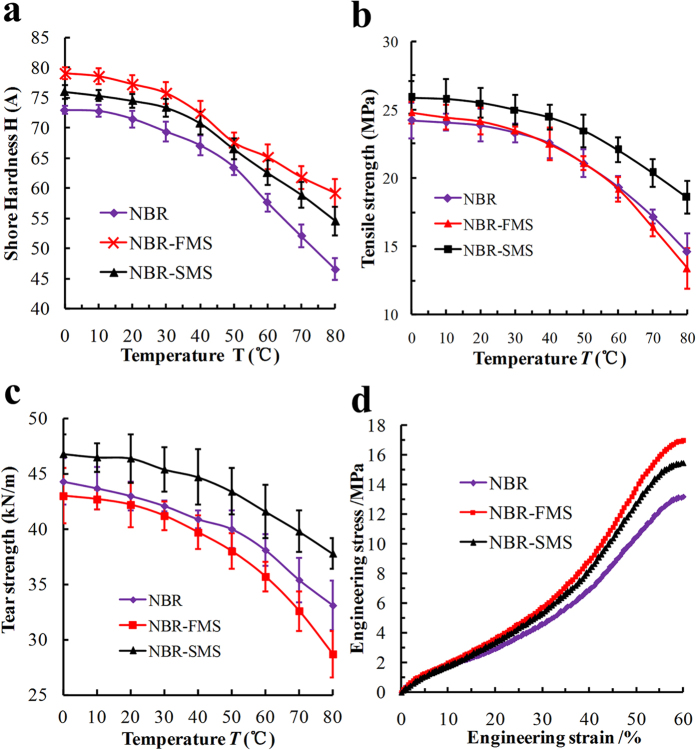
Typical mechanical property behaviours of the three rubber materials. Their (**a**) shore harnesses, (**b**) tensile strengths, (**c**) tear strengths under different temperature environments, and (**d**) their stress-compression curves at the compressive strain rate of 2640 s^−1^ and room temperature.

**Figure 3:**
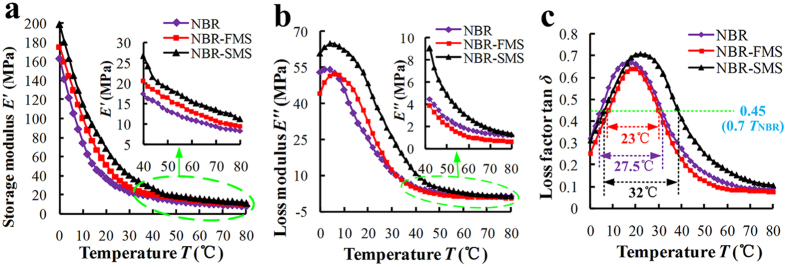
Trends in the (**a**) storage modulus, (**b**) loss modulus, and (**c**) loss factor of the three rubber materials under different temperature conditions.

**Figure 4:**
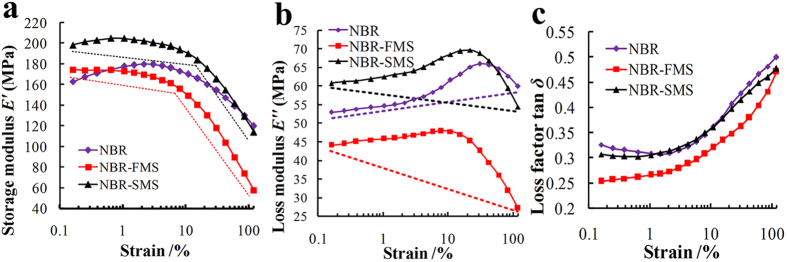
Payne effects of the (**a**) storage modulus, (**b**) loss modulus, and (**c**) loss factor for the three rubber materials under different strain amplitudes with a frequency of 10 Hz at room temperature (25 °C).

**Figure 5:**
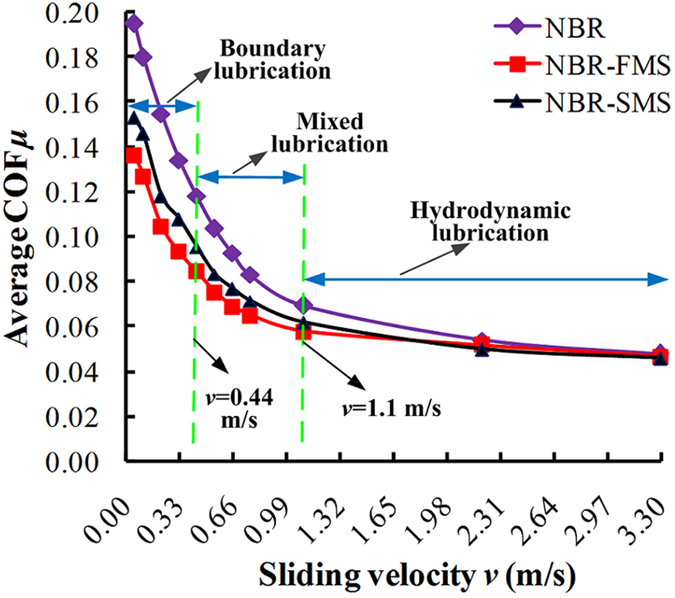
Effect of different MoS_2_ nanoparticles on the average COFs between the rubber and ZCuSn_10_Zn_2_ ring-discs.

**Figure 6:**
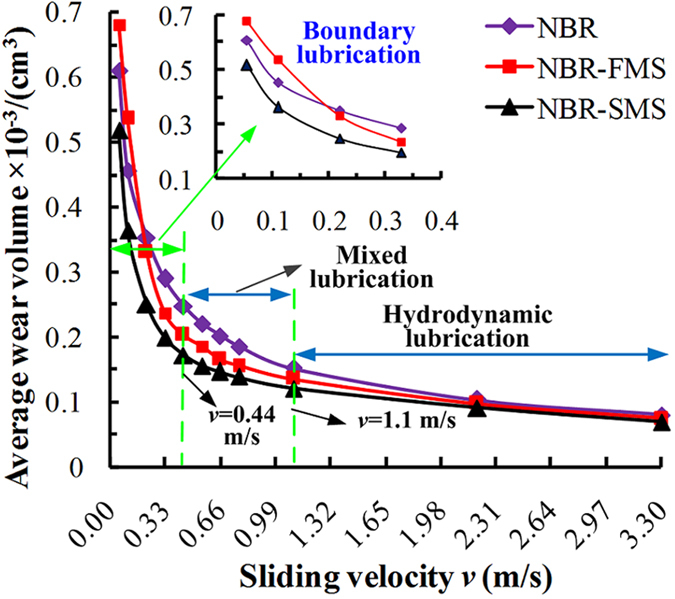
Effects of different MoS_2_ nanoparticles on the average distance wear volumes of the rubber ring-discs.

**Figure 7:**
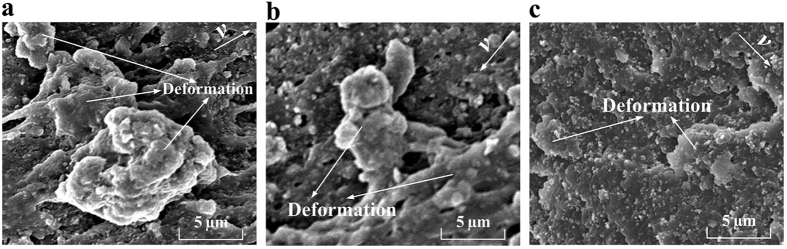
SEM images of the wear surfaces of tested NBR ring-discs at (**a**) 0.055, (**b**) 0.55, and (**c**) 3.3 m/s.

**Figure 8:**
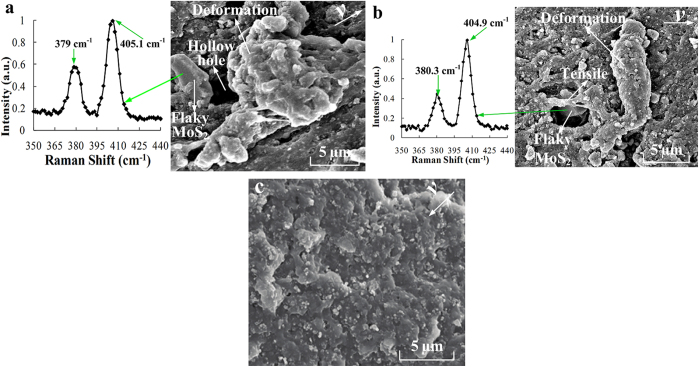
SEM images of the wear surfaces of tested NBR-FMS ring-discs at (**a**) 0.055, (**b**) 0.55, and (**c**) 3.3 m/s.

**Figure 9:**
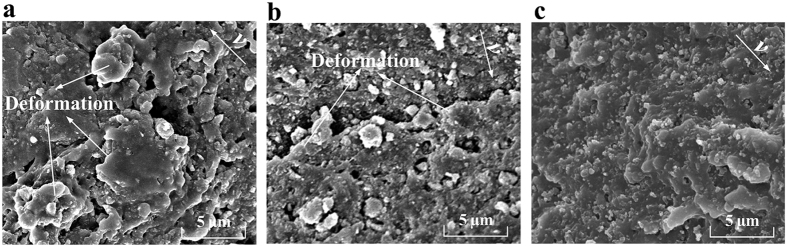
SEM images of the wear surfaces of tested NBR-SMS ring-discs at (**a**) 0.055, (**b**) 0.55, and (**c**) 3.3 m/s.

**Figure 10:**
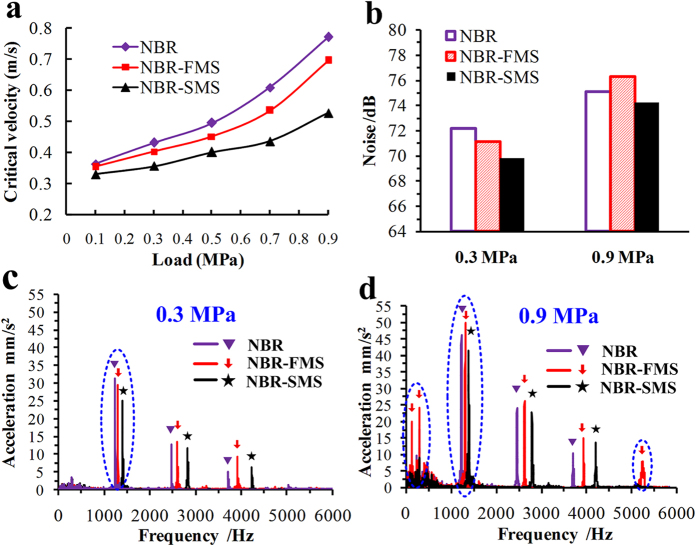
Frictional noise and vibration characteristics of the three rubber ring-discs at room temperature. (**a**) Critical velocities of the frictional vibration of the three rubber materials under different applied loads; (**b**) frictional noise of three rubber materials at 0.3, 0.9 MPa and 0.22 m/s; frequency-domain spectrum after filtering of three rubber materials at (**c**) 0.3, (d) 0.9 MPa and 0.22 m/s.

**Figure 11:**
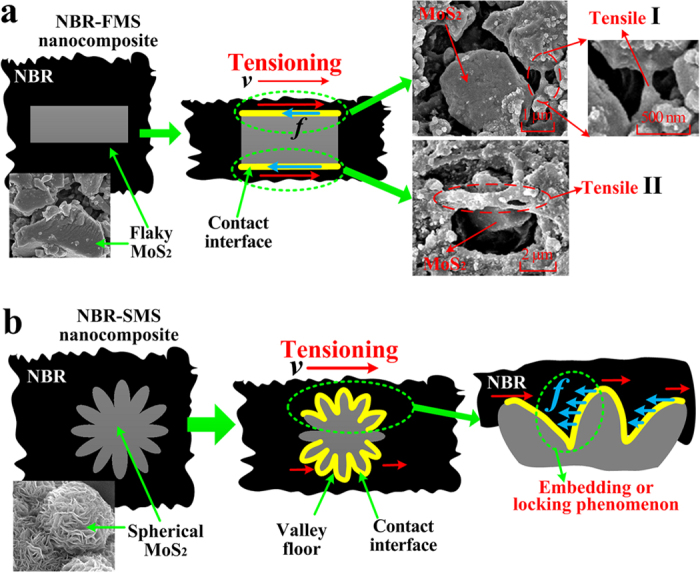
Tension models of (**a**) flaky MoS_2_, (**b**) spherical MoS_2_ nanoparticles and rubber material.

**Figure 12:**
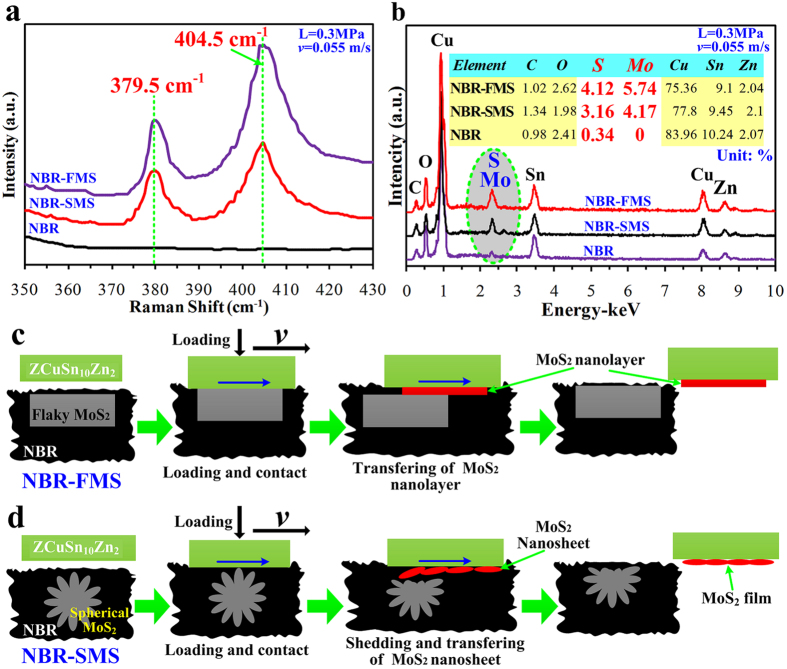
Lubrication mechanism models of the flaky and spherical MoS_2_ nanoparticles for rubber material. (**a**) Raman spectroscopy of the ZCuSn_10_Zn_2_ ring-discs’ wear surfaces tested against the three rubber materials at 0.055 m/s and 0.3 MPa; (**b**) EDS of the ring-discs’ wear surfaces; (**c**) lubrication mechanism model of the flaky MoS_2_ nanoparticles; (**d**) lubrication mechanism model of the spherical MoS_2_ nanoparticles.

**Figure 13:**
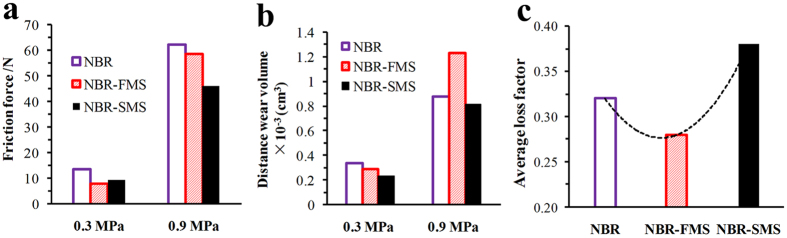
(**a**)Friction forces, (**b**) distance wear volumes of the three rubber materials under 0.3, 0.9 MPa and 0.22 m/s and (**c**) average loss factors of the three rubber materials in the range from 0 °C to 80 °C.

The flaky MoS_2_ nanoparticles’ surfaces appeared smooth (see the inset of Fig. 1a). The specific surface area was 3 ± 0.5 m^2^/g, and the sizes ranged between 1~6 μm. The higher-magnification SEM image in the inset of Fig. 1b provides a clear view of a microsphere’s surface morphology of MoS_2_ nanoparticles with diameters of approximately 500 nm. Many nanosheets were on the surface of the microsphere, thus making the surface rough and thereby increasing the specific surface area, which were as high as 100 ± 10 m^2^/g.

A NBR material with no MoS_2_ nanoparticles was used as a reference material. Two other types of NBR materials were added to the flaky and spherical MoS_2_ nanoparticle solid lubricants. Various ingredients were then added according to the formulas given in Table S1. The three rubber materials were made into the rubber ring-discs for the sliding tests (Fig. S1b), and also were made into dumbbell shaped specimens and trousers shaped specimens to study their tensile strength and tear strength properties. The materials were made into the cylinders (with a height of 2 ± 0.1 mm and a diameter of 10 ± 0.1 m) and rectangle specimens (their lengths, weights and heights were 2 ± 0.1 mm, 7 ± 0.1 mm and 50 ± 0.5 mm) for their compressive properties and dynamic mechanic analysis (DMA) respectively. ZCuSn_10_Zn_2_ was made into the ring-discs as the counterpart with an outer diameter of 46 mm and internal diameter of 38 mm as viewed in Fig. S1a.

### Wear tests

The sliding wear tests for the rubber ring-discs and ZCuSn_10_Zn_2_ ring-discs were conducted on a commercial disc-on-disc wear testing machine (CBZ-1 tribo-tester, Haima Ltd., China) under water-lubricated condition, which is illustrated schematically in Fig. S1c. During the tests, the lower rubber ring-disc specimen remained stationary while the upper ZCuSn_10_Zn_2_ ring-disc specimen slid against the rubber ring-disc specimen’s surface with a rotational motion. The sliding velocities were 0.055, 0.11, 0.22, 0.33, 0.44, 0.55, 0.66, 0.77, 1.1, 2.2 and 3.3 m/s, and the nominal pressure used was 0.3 MPa which was the normal service load of the ship water-lubricated stern tube rubber bearing. The normal load (0.3 MPa) and high load (0.9 MPa) were chosen to investigate the effects of the flaky and spherical MoS_2_ nanoparticles on the frictional noise, and the velocity was set to 0.22 m/s. A real-time sound pressure spectrum analysis system (CRY2120U, CRY Sound Co., Ltd, China) was used to collect the frictional noise as show in Fig. S1. The duration of each test was 8 hrs to ensure the stability of the wear mass loss, and was repeated twice under the same condition to check the repeatability of the results. The COFs were measured every 3 seconds during the wear tests.

BK Pules Acoustic and Vibration Measurement System were used to collect and analyze the vibration characteristics as shown in Fig. S1d. The decreasing velocity method was used to search for the critical velocities of frictional vibration. The nominal pressure used was set to be 0.1, 0.3, 0.5, 0.7 and 0.9 MPa. First, the rotational speed was set to be 500 r/min (1.1 m/s) for the rubber ring-discs and ZCuSn_10_Zn_2_ ring-discs under one load condition. The sliding velocity was so large that no frictional vibration was generated under the tested load conditions. Next, the rotational speed was decreased by 5 r/min (0.011 m/s) without stopping, and steady rotation was continued for 5 min. The vibration signals were collected online. If abnormal vibration phenomena did not appear, the rotational speed was decreased by 5 r/min until the critical velocities of the frictional vibration were identified.

### Characterization and analysis

The shore hardness of the rubber ring-discs was measured using an automatic electronic hardness tester (Digi test II, Germany). The tensile strength and tear strength properties of the three rubber material were examined using a high and low temperature tensile tester (UTM-GD Tester, Shanghai Youhong Testing Instrument Co. Ltd., China). The compressive property was measured using a SHPB experiment system (National University of Defense Technology, China) at the strain rate of 2640 s^−1^ and room temperature. The storage modulus and loss modulus were measured using a dynamic thermomechanical analyzer (DMAQ800, TAInstrumentsCorp., USA) with three point bending test method under the tension mode with a frequency of 10 Hz, and the strain amplitude ranging from 0 °C to 80 °C was 20 μm. The surface topographies of the tested rubber ring-discs and EDS analysis of the ZCuSn_10_Zn_2_ ring-discs’ wear surfaces were examined using a JSM-6701F scanning electron microscope (manufactured by JEOL in Japan). Raman spectra with the resolution of 0.7 cm^−1^ were obtained to identify the MoS_2_ nanoparticles. X-ray diffraction patterns were obtained on a Bruker D8 Advance XRD machine to analyze the crystalline phase features of three rubbers. The surface roughness was measured using laser-interference profilometry (LI–3, Huazhong University of Science and Technology, China).

The wear mass losses of the rubber ring-discs were determined by measuring the weights before and after the tests, using an analytical balance with a resolution of 0.00000l g (MS205DU, Shanghai Jiehui Electronic Technology Ltd., China). The tested rubber ring-disc specimens before weighing were ultrasonically cleaned in water and dried for 48 hrs in an oven at 40°. The distance wear volumes of the three rubber materials, which meant the wear volumes under the sliding distance of 1 km, were chosen to investigate the effects of the sliding velocity and MoS_2_ nanoparticles during wear progress, which were calculated using the Eq. (1):


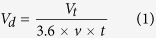


where *V*_*t*_ is the total wear volumes, mm^3^, *v* is sliding velocity, m/s, and *t* is the sliding time, h. The average value of distance wear volumes of each type of rubber ring-discs under the same condition was presented as the wear volumes loss result.

## Additional Information

**How to cite this article**: Dong, C. *et al*. Tribological Properties of Water-lubricated Rubber Materials after Modification by MoS_2_ Nanoparticles. *Sci. Rep*. **6**, 35023; doi: 10.1038/srep35023 (2016).

## Supplementary Material

Supplementary Information
