# Supplementary material for: Tribological Properties of Water-lubricated Rubber Materials after Modification by MoS2 Nanoparticles
Source: Sci Rep. 2016 Oct 7;6:35023. doi: 10.1038/srep35023 (PMC5054368; doi:10.1038/srep35023)
Supplement: Supplementary Information [file srep35023-s1.pdf]

Tribological Properties of Water-lubricated Rubber Materials after Modification by MoS_2 Nanoparticles

Conglin Dong[1, 2], Chengqing Yuan[1, 2,*], Lei Wang[3], Wei Liu[3], Xiuqin Bai[1, 2], Xinping Yan[1, 2]

[1]Key Laboratory of Marine Power Engineering & Technology (Ministry of Transport), Wuhan University of Technology, Wuhan 430063, P.R. China
[2] Reliability Engineering Institute, National Engineering Research Center for Water Transport Safety, Wuhan University of Technology, Wuhan 430063, P.R. China
[3]China Ship Development and Design Center, Wuhan 430064, P.R. China
*Corresponding. ycq@whut.edu.cn (C.Q. Yuan); Fax: +86-27-86549879; Tel: +86-27-86554969.

Supplementary information

Various ingredients were then added according to the formulas given in Table S1. The formulations were chosen to manufacture rubber specimens to be as similar as possible to the typically manufactured formulations with the addition of ingredients and different types of MoS_2 nanoparticles. Thus, the viscosity and consistency of the formulas were adequate for commercial processing routes and allowed the effects of the addition of MoS_2 nanoparticles to be explored[1]. The combinations were mixed in a rubber and plastic mixing device (LH-60, Kechuang Rubber and Plastic Machinery Factory, Shanghai, China). Mixing was conducted at 90°C for 13 min in an open two-roll laboratory mixing mill (Ø160×320, Shanghai Rubber Machinery Factory, China); this process was repeated 20 times to achieve complete mixing at room temperature. The optimum cure time (T90) was determined by a rubber curometer (Youshen Electronic Instrument Company, Beijing, China). The vulcanization of the specimens was conducted using a compression moulding press (Hangfa Hydraulic Engineering Factory, Chengdu, China) at 15MPa and 170°C for T90, and three types of rubber material were obtained.

Table S1. Formulas of the three types of rubber material.

Materials	Amounts (phr[a])		
	NBR	NBR-FMS	NBR-SMS
NBR	100	100	100
Flaky MoS_2		8	
Spherical MoS_2			8
Carbon blacks (N772)	40	40	40
Sulfur	2	2	2
ZnO	5	5	5
Anti-aging reagent (4010NA)	1.0	1.0	1.0
Coupling agent (kh550)	1.5	1.5	1.5
Hexamethylenetetramine	3.5	3.5	3.5
Stearic acid (SA)	1	1	1

[a]Parts by weight per hundred parts of rubber materials.

The three rubber materials were made into the rubber ring-discs for the sliding wear tests (Fig. S1(a)). Their outer diameter, internal diameter, thickness and cross-sectional area (*S*) of were 44.5 mm, 39.5 mm, 8 mm and 32.97×10^{-5} m^2, respectively. Their mean surface roughness (*S*a) was 0.6±0.1 μm. Gain the excellent anti-corrosion capability, the $ZCuSn_{10}Zn_2$ sleeve is rotated by the stern shaft and slid on the surface of rubber stern tube bearing under the water-lubricated condition. Therefore, $ZCuSn_{10}Zn_2$ was made into the ring-discs as the counterpart with an outer diameter of 38 mm and internal diameter of 46 mm. Its thickness and cross-sectional area were 10 mm and 52.752×10^{-5} m^2. Its surface roughness (*S*a) was 0.5 ±0.1 μm. The main element proportions and important mechanical properties of $ZCuSn_{10}Zn_2$ are displayed in Tables S2 and S3, respectively.

Table S2. The main element proportions of $ZCuSn_{10}Zn_2$

Cu	Sn	Zn	Pb	Ni	Impurity
The other	9.0～11.0	1.0～3.0	⩽1.5	⩽2.0	⩽1.5

Table S3. Important mechanical properties of $ZCuSn_{10}Zn_2$.

Brinell hardness HB	Young's modulus E (GPa)	Shear modulus G (GPa)	Mass density*ρ* (g/cm^3)	Tensile strength (MPa)	Yield strength (MPa)	Elongation δ5 (%)
⩾785	108	39	8.738	⩾245	⩾140	40

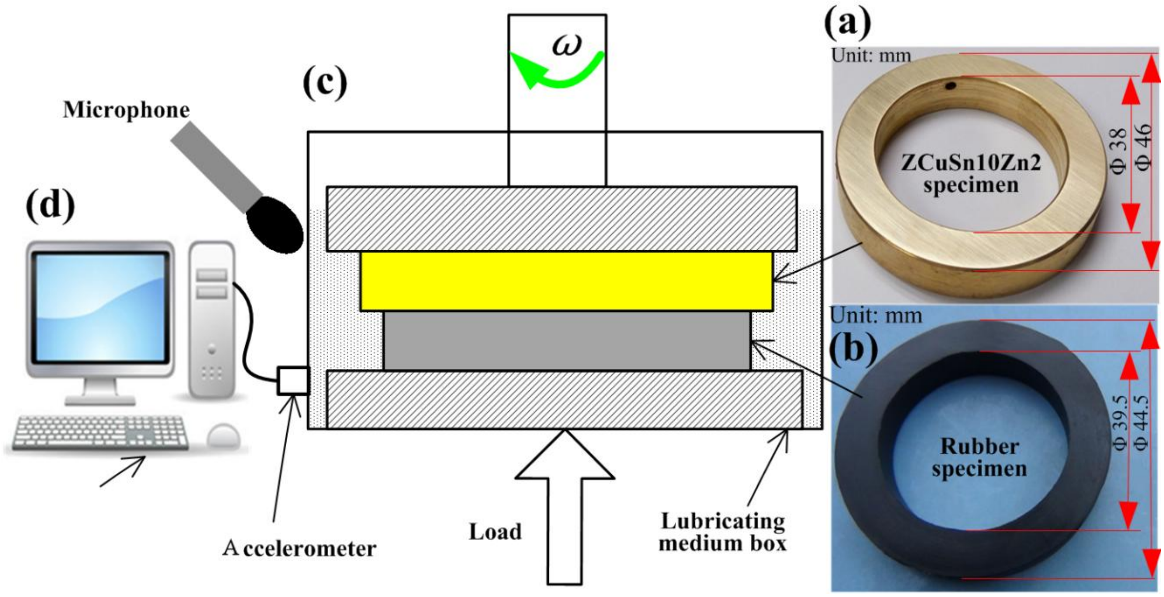

Figure S1. Schematic diagram of CBZ-1 tribo-tester used in this study.

References

1. Mostafa, A., Abouel-Kasem, A., Bayoumi, M. R., El-Sebaie, M.G. The influence of CB loading on thermal aging resistance of SBR and NBR rubber compounds under different aging temperature. *Mater. Design* **30**, 791–795 (2009).
